#  HLA-E–restricted T cells primed by a modified HLA-B*57:01–restricted HIV-1 peptide suppress HIV-1 replication

**DOI:** 10.1172/jci.insight.203593

**Published:** 2026-05-19

**Authors:** Hong Sun, Hongbing Yang, Max N. Quastel, Simon Brackenridge, Wanlin He, Anna E. Kliszczak, Margarida Rei, Persephone Borrow, Geraldine M. Gillespie, Andrew J. McMichael

**Affiliations:** 1 Centre for Immuno-Oncology, Nuffield Department of Clinical Medicine, University of Oxford, Oxford, United Kingdom.; 2 Chinese Academy of Medical Sciences Oxford Institute, Oxford, United Kingdom.; 3 State Key Laboratory for Diagnosis and Treatment of Infectious Diseases, NHC Key Laboratory of AIDS Prevention and Treatment, National Clinical Research Center for Laboratory Medicine, The First Hospital of China Medical University, China Medical University, Shenyang, China.; 4 State Key Laboratory of Oral Diseases and National Center for Stomatology and National Clinical Research Center for Oral Diseases, West China Hospital of Stomatology, Sichuan University, Chengdu, China.; 5 Gulbenkian Institute for Molecular Medicine, Lisbon, Portugal.; 6 Faculdade de Medicina, Universidade de Lisboa, Lisbon, Portugal.

**Keywords:** AIDS/HIV, Immunology, Adaptive immunity, MHC class 1, T cells

## Abstract

HLA-E–restricted HIV-specific T cells offer exciting possibilities for immunotherapy. However, HLA-E binding peptides are rare. A recent study showed that in HLA-B*57:01–positive people with HIV, the peptide that dominates the T cell response, KAFSPEVIPMF (KF11), also stimulates HLA-E–restricted T cells, even though direct binding of this peptide to HLA-E could not be demonstrated. We therefore changed position 2 alanine for methionine in the peptide (referred to as KMF11), which greatly enhanced binding to HLA-E. This enabled the generation of stabilized HLA-E-KMF11 tetramers, which were used to select and then grow specific T cell clones from T cells of HLA-B*57:01–negative blood donors primed with this peptide in vitro. Approximately 20% of these T cell clones reacted with HLA-E–positive cells presenting the native KF11 peptide. Furthermore, these T cells inhibited replication of HIV-1 NL4-3 in CD4^+^ T cells in vitro. Therefore, this native peptide can be presented by HLA-E to CD8^+^ T cells, although priming in vivo may depend on cross-reactivities to classical MHC-Ia types. Nevertheless, such T cells could be exploitable for immunotherapy given the conservation of this HIV-1 peptide epitope and the non-polymorphism in HLA-E.

## Abstract 2

HLA-E, which is the same in all people, binds a small fragment of an HIV protein and stimulates the immune response to destroy infected cells.

## Graphical abstract

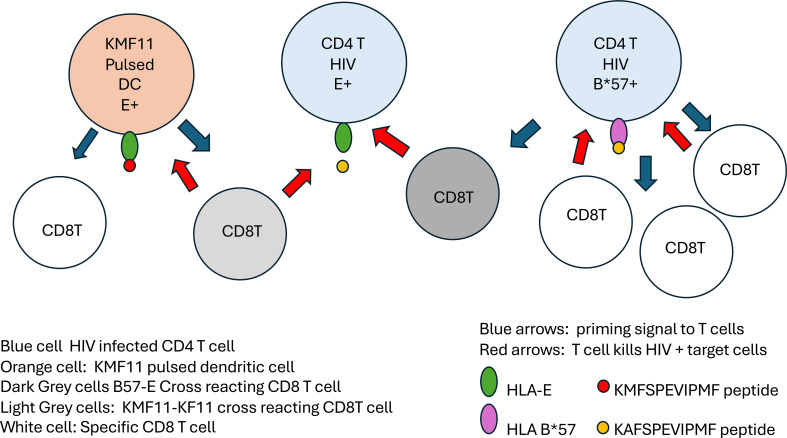

## Introduction

As a nonclassical HLA-Ib molecule, HLA-E is primarily notable for presenting the signal nonamer peptide, typically VMAPRTLVL (VL9), derived from conventional HLA-A and -C, plus some -B molecules, to the CD94/NKG2A/C receptor family expressed by NK cells and a subset of CD8^+^ T cells. The interactions with the higher affinity inhibitory receptor NKG2A-CD94 and the lower affinity activating receptor NKG2C-CD94 play a crucial role in regulating innate immunity and maintaining immune homeostasis (1).

Recent increasing evidence suggests that HLA-E can play a secondary and sometimes pivotal role in adaptive immunity, by presenting pathogen-derived peptides to CD8^+^ T cells with potential to combat invasive infections. For instance, during *Mycobacterium tuberculosis* infection, CD8^+^ T cells have been shown to recognize multiple pathogen-derived peptides presented by HLA-E, engaging in the immune response to *M*. *tuberculosis* infection and complementing conventional MHC-I responses (2–4). Similarly, HLA-E–restricted CD8^+^ T cell responses specific to human CMV, hepatitis B virus, and SARS-CoV-2 have been described in viral infections though usually at lower levels than classical T cell responses (5–7). Notably, unlike classical HLA-I molecules, HLA-E appears relatively resistant to virus-mediated downregulation (7–9) and is characterized by its limited genetic polymorphism and near-ubiquitous expression on the cell surface, albeit at low levels (10). These features make HLA-E an attractive target for CD8^+^ T cell–mediated immune therapy across diverse infections.

Despite significant advancements in antiviral drug therapy, HIV/AIDS remains an incurable and life-threatening disease, with approximately 40 million people living with HIV, with 1.3 million new infections and 630,000 deaths reported in 2023 (11). These statistics highlight the continuing need for inexpensive, effective vaccines. The failure of drug therapy to eradicate the virus from people living with HIV-1 highlights a need for novel approaches, including immunotherapies, to achieve cures. Current vaccine efforts have predominantly focused on generating neutralizing antibodies, but with increasing recognition that classical MHC-Ia–restricted T cell responses might also protect synergistically (12–14). The recent recognition that HLA-E can utilize an alternative antigen presentation pathway may provide new avenues for vaccine and therapeutic development (10). Remarkably, Hansen et al. demonstrated that a rhesus CMV-vectored SIV (RhCMV/SIV strain 68-1) vaccine conferred protection to over 50% of rhesus macaques against a highly pathogenic SIV challenge (15). This unprecedented level of protection was mediated by CD8^+^ T cells recognizing SIV peptides presented by Mamu-E, the rhesus ortholog of HLA-E (16), providing a compelling rationale for leveraging HLA-E–restricted immunity in human vaccine strategies. Furthermore, the priming of MHC-E–restricted T cells can be achieved in other primate species, such as cynomolgus macaques, but does require species-matched CMV vectors (17).

We previously identified an HLA-E–presented HIV Gag-derived peptide, RMYSPTSIL (RL9HIV), which is homologous to the SIV RMYNPTNIL (RL9SIV) previously identified as an immunodominant supertope in rhesus macaques immunized with the RhCMV-SIV vaccine (18). We demonstrated that RL9HIV could elicit HLA-E–restricted CD8^+^ T cell responses capable of effectively controlling HIV infection in human CD4^+^ T cells in vitro (18). In a later study, we identified a second HLA-E binding peptide in HIV-1 Rev (residues 100–108), that primed CD8^+^ T cell clones that effectively suppressed HIV-1 replication, also in vitro (19).

A recent study in people with HIV who were positive for HLA-B*57 identified CD8^+^ T cell responses targeting the HIV-derived peptide KAFSPEVIPMF (KF11), which exhibited HLA-E restriction alongside its better-known HLA-B*57:01 restriction (20). The latter T cell response, alongside additional Gag-specific T cell responses, is associated with control of HIV infection (21, 22). The KF11-specific HLA-E–restricted T cell responses were detected in vitro using PBMCs derived from people with HIV who had HLA-B*57:01, cocultured with genetically modified cell lines expressing specific HLA antigens. However, it was not possible to detect HLA-E-KF11 multimer binding and direct suppression of HIV-infected cells was not reported.

In this study, we explore HLA-E–restricted KF11-specific T cells further. To overcome the difficulty of demonstrating binding of the KF11 peptide to HLA-E, we substituted alanine with methionine at position 2 of KF11, a preferred anchor for HLA-E binding that is deeply buried in the B pocket (23). This modification, referred to as the KMF11 peptide, significantly enhanced HLA-E binding, as demonstrated using multiple optimized HLA-E binding assays. The resulting KMF11/HLA-E tetramers were then used to select and expand KMF11-specific T cell clones after priming HLA-B*57:01–negative blood donors in vitro. Using antigen recognition and coculture suppression assays, we observed that a number of T cell clones exhibited cross-reactivity with the native KF11 peptide– and HLA-E–expressing antigen-presenting cells. Moreover, we identified 4 T cell receptors (TCRs) capable of inhibiting HIV-1 NL4-3 virus replication in primary CD4^+^ T cells in vitro. These findings help pave the way toward vaccine and immunotherapy strategies aimed at targeting protective HLA-E–restricted CD8^+^ T cell responses.

## Results

### A mutant peptide of HIV Gag KF11 binds robustly to HLA-E molecule.

**Figure 1:**
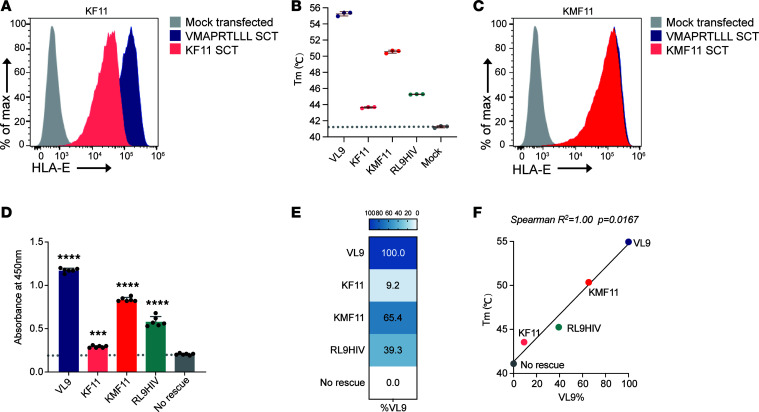
Identification of a mutant peptide of HIV Gag-derived KF11 as an HLA-E binding peptide. (**A**) Binding of the HIV Gag_162-172_ KAFSPEVIPMF (KF11) to HLA-E was evaluated using the single chain trimer (SCT) expression assay with VMAPRTLLL (VL9) included as a positive control. (**B**) The thermal melt (Tm) values of peptide-free HLA-E-β2m complexes incubated with 10 M excess of KF11 and the peptide position 2 alanine to methionine variant peptide (KMF11) was assessed by nano-differential scanning fluorography (nano-DSF). The positive control VL9 and mock no-peptide control were included for reference. The dot plot shows 3 biological replicates per peptide, presented as mean ± SD. Two technical replicates per peptide were measured per run. (**C**) Flow cytometry analysis of SCT expression demonstrated the binding potential of the mutant peptide KMF11 compared with the VL9 positive control. (**D**) HLA-E binding to KF11 and KMF11 were subsequently assessed using the peptide-exchange HLA-E peptide binding ELISA (25). The bar chart illustrates the raw absorbance value at 450 nm (*y* axis) of tested peptides, including KF11, KMF11, RL9HIV, positive control VL9, and no-rescue negative control, which included the same concentration of DMSO used for the test peptides (*x* axis). Data shown as mean ± SD. Statistical significance was assessed using 1-way ANOVA with Dunnett’s multiple-comparison test. ****P < 0.0001. For ELISA-based screens, 3 independent peptide exchange reactions were performed per individual peptide (*n* = 3), with 2 technical replicas per peptide tested. (**E**) The heatmap denotes the ranking of HLA-E binding strength data obtained using the sandwich ELISA, indicated as percentage of VL9 binding. (**F**) Correlation of peptide-exchange HLA-E peptide binding ELISA reads and nano-DSF assay Tm data using Spearman’s correlation method. VL9, blue; KF11, pink; KMF11, red; RL9HIV, green; no-rescue, gray.

An immunodominant HLA-B*57:01–restricted epitope, HIV Gag_162-172_ KAFSPEVIPMF (KF11), was recently reported to also be presented by HLA-E*01:01 (20). In that study, a genetically modified HLA-E–expressing 721.221 cell line was peptide-pulsed and shown to be recognized by T cells from a subset of patients with HIV after coculture. However, these HLA-E–restricted T cell responses were predominantly detected in HLA-B*57–positive donors, and it was not possible to visualize the T cells directly using KF11/HLA-E multimers. To confirm and extend this finding, we first constructed single-chain peptide-β2-microglobulin (β2m)–HLA-E (HLA-E*01:03) trimers (SCTs) and transfected them into HEK293T cells to assess whether KF11 could stabilize and enhance the cell surface expression of HLA-E detected by flow cytometry. The result showed that there was detectable binding of KF11 to HLA-E when stabilized in the SCT format (Figure 1A). We further evaluated the binding of KF11 to HLA-E*01:03 using differential scanning fluorimetry (DSF), which measures the melting temperature (T_m_) of the peptide–HLA-E complex (pHLA-E). Since we can generate peptide-free HLA-E-β2m complexes (7), we were able to directly add excess peptide to these complexes. At a 10 M excess peptide concentration over HLA-E, the T_m_ of WT KF11 bound to HLA-E was 43.7°C, which was only slightly higher than the no-rescue peptide control at 41.3°C. This indicates very weak binding to HLA-E. The positive finding with the SCT probably reflects the tethering of the peptide to the HLA-E molecule, enabling peptide to rebind repeatedly after dissociation.

Given that the preferred amino acid anchor residue of HLA-E at position 2 is methionine (M), which is buried in the HLA class I B pocket (24), we substituted this amino acid for the position 2 alanine in KF11 to give the peptide KMFSPEVIPMF (KMF11). This mutant peptide dramatically increased binding to HLA-E (T_m_ = 50.6°C), compared with the WT KF11 peptide, and approached that of the dominant natural signal peptide VL9 (Figure 1B). An SCT assay incorporating the KMF11 peptide was subsequently performed and demonstrated that KMF11 stabilized HLA-E expression to a level comparable to that of the VL9 peptide (Figure 1C).

To further confirm these findings, we next tested binding of KF11 and KMF11 to HLA-E using a peptide-exchange ELISA, as previously described, with the HLA class I signal peptide VL9 as a positive control, and with a no-rescue peptide control using the same concentration of DMSO as for the tested peptide (18, 25). Very weak binding of KF11 to HLA-E was observed, amounting to 9.2% of the VL9 signal, and much lower than for the RL9HIV peptide (39.3% VL9). That level of binding of the RL9HIV peptide approached the minimum required for stable HLA-E tetramers and needs further stabilization strategies to generate reliable reagents (18) (Figure 1, D and E). In line with nano-DSF and SCT data, the mutant KMF11 dramatically increased binding, equating to 65.4% of the VL9 control (Figure 1E). The binding in the ELISA, measured as a percentage of VL9 binding, was highly correlated with the T_m_ result from the previous nano-DSF assay (Spearman’s correlation test *R*^2^ = 1.00, *P* = 0.0167, Figure 1F). Therefore, these data collectively indicate that the mutant peptide KMF11 binds strongly to HLA-E in comparison to the weakly binding WT KF11 peptide.

Because the side chain of the second amino acid in HLA-E binding peptides is buried in the B pocket of HLA-E (24), we propose that stabilized KMF11/HLA-E tetramers could be used to screen native KF11-specific TCRs with a good chance of detecting cross-reactive, KF11-specific T cells. This approach could offer a means of selecting these T cells for functional evaluation.

### Generation of KMF11-specific HLA-E–restricted CD8^+^ T cells from primed T cells from healthy donors.

**Figure 2:**
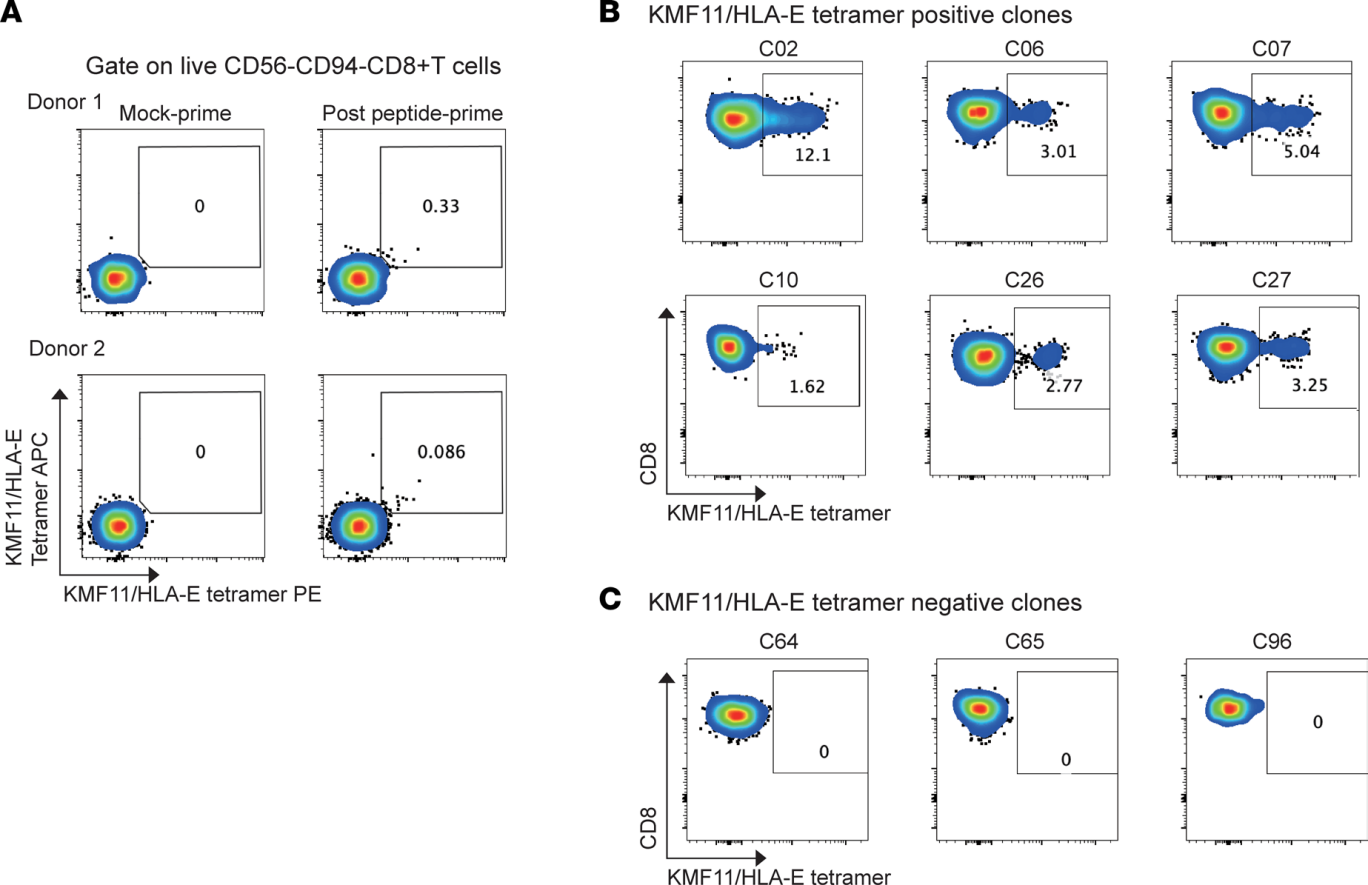
Generation of KMF11-specific HLA-E–restricted CD8 cell clones using an in vitro DC-differentiated T cell–priming assay. (**A**) KMF11-specific HLA-E–restricted CD8^+^ T cells were primed from healthy donor–derived PBMCs using a DC differentiation T cell–priming protocol (7, 26). Representative flowcharts indicate the KMF11/HLA-E tetramer staining of primed T cells where tetramers were conjugated with allophycocyanin or phycoerythrin fluorescence, with mock-primed (left) and peptide-primed (right) conditions shown. Double tetramer^+^ gates were set on CD56 and CD94 double-negative CD8^+^ T cells. (**B** and **C**) KMF11/HLA-E dual tetramer^+^ CD8^+^ T cells were sorted using a FACSAria sorter, then seeded at 0.4 cells per well and cultured with irradiated feeder cells (45 Gy) in complete media with PHA/IL-2 for 12 days. CD8^+^ T cell clones were identified if KMF11/HLA-E tetramer staining showed a distinct population (**B**) compared with other clones that showed no staining (examples in **C**).

We next tested whether KF11-specific HLA-E–restricted CD8^+^ T cells could be primed in vitro using the KMF11 peptide. We used PBMCs from 2 HIV-1–negative, HLA-B*57:01–negative blood donors (Supplemental Table 1; supplemental material available online with this article; https://doi.org/10.1172/jci.insight.203593DS1). T cells were primed with KMF11 at a concentration of 50 μM plus a cocktail of cytokines to activate and maintain the specific T cells in an autologous DC differentiation T cell–priming assay in vitro (18, 26). After 8 days of culture, we costained the cells with KMF11/HLA-E tetramers conjugated to allophycocyanin and phycoerythrin and gated on double-positive cells to reduce the chances of isolating nonspecific cells. We also excluded CD56-positive and CD94-positive CD8^+^ T cells to avoid contamination by NK cells or CD94/NKG2x-expressing T cells. After 8 days of peptide priming, KMF11/HLA-E–staining tetramer cells were detected at the frequency of 0.33% of CD8^+^ T cells in donor 1 and 0.086% in donor 2 (Figure 2A).

In order to dissect the functionalities of KMF11-specific HLA-E–restricted CD8^+^ T cells, we generated KMF11-specific CD8^+^ T cell clones by FACS of tetramer-positive CD8^+^ T cells. Sorted cells were cultured at fewer than 0.4 cells per well in microwell plates with irradiated allogeneic feeder cells, phytohemagglutinin (PHA), and IL-2, as previously described (7). After 12 days, a total of 586 clones proliferated, and KMF11/HLA-E tetramer staining identified KMF11-specific T cell clones C02, C06, C07, C10, C26, and C27, with distinct HLA-E tetramer–staining patterns, as shown in Figure 2B, similar to our previous observation for SARS-CoV-2–specific T cell clones (7). The relatively low frequencies of tetramer staining of these cloned T cells likely reflects the low-affinity binding of their TCRs, similar to the patterns we have observed previously for other HLA-E–restricted peptide epitopes (7, 18). Several clones gave no distinct tetramer staining, suggesting they are probably bystander activated T cells, and these clones served as negative controls (Figure 2C).

TCRs for all positive and negative HLA-E-KMF11 binding and nonbinding clones, including those identified in Figure 2, were full-length sequenced using the SMART (Switching Mechanism at 5′ end of RNA Template) and 5′RACE (5′Rapid Amplification of cDNA Ends) technique. Each clone expressed a single TCRβ chain, and the nearly 100% sequence read identities confirmed that they were clonal expansions (Table 1). C07 expressed 2 TCRα chains.

The 6 clones C02, C06, C07, C10, C26, and C27 were further screened using specific monoclonal antibodies for expression of coreceptors that could possibly account for HLA-E-KMF11 tetramer binding: NKG2A/C, ILT-2, and ILT-4. None of the clones expressed these receptors, and none stained with HLA-E tetramers containing the VL9 peptide ligand of the NKG2A/C-CD94 receptors, indicating that KMF11 tetramer binding was specific to their TCRs (Supplemental Figure 1, A and B).

### Antigen recognition of KMF11-specific HLA-E–restricted CD8^+^ T cells.

For functional analysis of the CD8^+^ T cell clones, stable HLA-E-KF11 expression on antigen-presenting cells was required. The HLA-Ia–deficient K562 chronic myelogenous leukemia cell line was therefore transduced with the construct expressing an SCT of peptide-β2m-HLA-E*01:03 heavy chain, giving a relatively stable HLA-E–peptide complex. First, constructs with SCTs incorporating the KF11 and KMF11 peptides, β2m, and the HLA-E*01:03 heavy chain were produced. A K562 line transduced with a construct expressing the SARS-CoV-2 peptide VMPLSAPTL (ECOV2P1), a strong binder to HLA-E (7), was also generated as a negative control. The sequences of the constructs were verified by Sanger sequencing. The different HLA-E–expressing K562 cells (K562E_KF11, K562E_KMF11, and K562E_ECOV2P1) were enriched by FACS and further validated by HLA-E antibody (clone: 3D12) staining by flow cytometry (Supplemental Figure 2).

**Figure 3:**
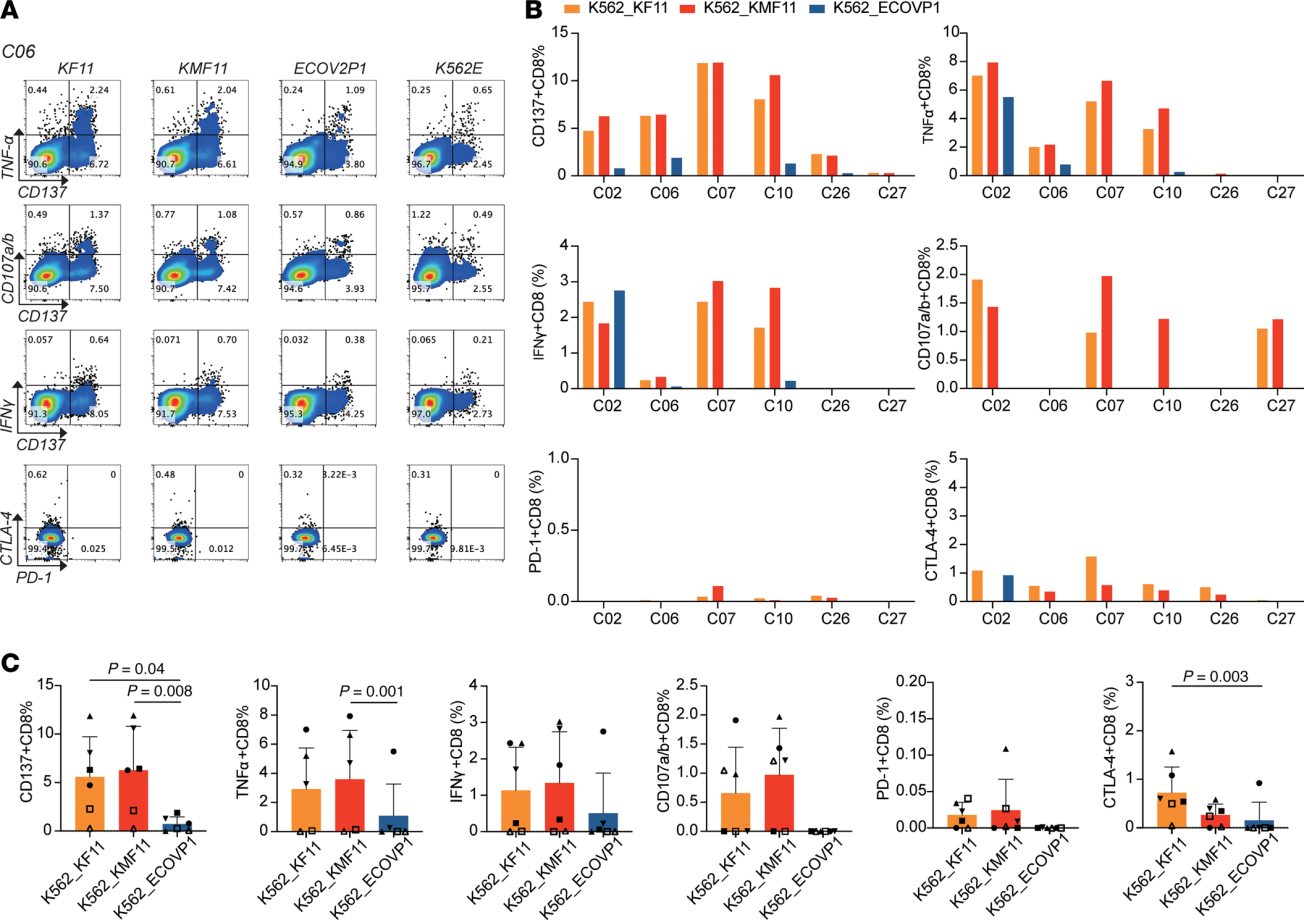
Functional characterization of HLA-E–restricted CD8^+^ T cell clones in response to antigen-presenting cells. (**A**) Representative flow cytometry plots of KMF11-primed clone C06, demonstrating cytokine production (TNF-α, IFN-γ), activation (CD137, CD107a/b), and inhibitory marker expression (PD-1, CTLA-4) after coculture with K562E_KF11, K562E_KMF11, and K562E_ECOV2P1 for an incubation period of 10 hours. HLA-E–expressing K562 cells (K562E cells) were included as negative controls. Intracellular cytokine staining of the above markers was performed after surface staining of Live/Dead Fixable Aqua viability dye, and anti-CD3 and anti-CD8 antibodies. Percentages of CD8^+^ T cells expressing the indicated markers are shown. (**B**) The expression of CD137, TNF-α, IFN-γ, CD107a/b, PD-1, and CTLA-4 for each CD8 clone in response to KF11-, KMF11-, and ECOV2-expressing K562E cells is shown. For normalization, the background of K562E control was subtracted from each antigen-specific condition. The *y* axis denotes the percentage of effector marker–positive CD8^+^ T cells, and the *x* axis denotes individual CD8^+^ T cell clones. (**C**) The expression of CD137, TNF-α, IFN-γ, CD107a/b, PD-1, and CTLA-4 expression in response to KF11-, KMF11-, and ECOV2P1-expressing K562E cells is noted in bar chart format. Friedman’s test was used to test significance. *P* values are indicated.

The functionality of the 6 KMF11-specific CD8^+^ T cell clones, C02, C06, C07, C10, C26, and C27, was measured by assessing expression of TNF-α, IFN-γ, CD107a/b, the activation molecule CD137, and inhibitory molecules programmed cell death 1 (PD-1) and cytotoxic T lymphocyte antigen 4 (CTLA-4) after coculture with K562E cell lines expressing different HLA-E–peptide SCTs. K562 cells expressing HLA-E only served as the mock control. Coculture with K562 cells expressing HLA-E only and K562E_ECOV2P1 cells stimulated very low levels of activation or cytokine release in the T cell clones, while HLA-E-KF11 and HLA-E-KMF11 upregulated expression of the activation markers (Figure 3A). The 6 selected T cell clones responded to both K562E_KMF11– and K562E_KF11–expressing antigen-presenting cells. Four of the clones showed elevated expression of CD137, whereas expression was low on C26 and clone C27 was negative. Clones C02, C07, and C10 produced relatively strong TNF-α and IFN-γ responses, though C02 gave similar responses to the ECOV2P1 negative control. CD107a/b expression was strongest on clones C02, C07, and C27 (Figure 3, B and C). All these clones expressed very low levels of PD-1 and CTLA-4 after stimulation (Figure 3, B and C).

To summarize, the 6 clones gave low but significant responses when stimulated by K562 cells expressing single-chain HLA-E KF11 and KMF11 trimers. There was no significant difference between KF11 and KMF11, although as noted above, the tethering of peptide to HLA-E may mitigate the very low binding affinity of KF11 to HLA-E. Clones C07 and C02 gave the strongest responses; however, C02 showed cross-reactivity to the ECOV2P1 peptide–HLA-E complex specifically in relation to TNF-α and IFN-γ production.

### Antigen-specific reduction of both KMF11- and KF11-SCT–expressing K562E cells by KMF11-specific HLA-E–restricted CD8^+^ T cells.

**Figure 4:**
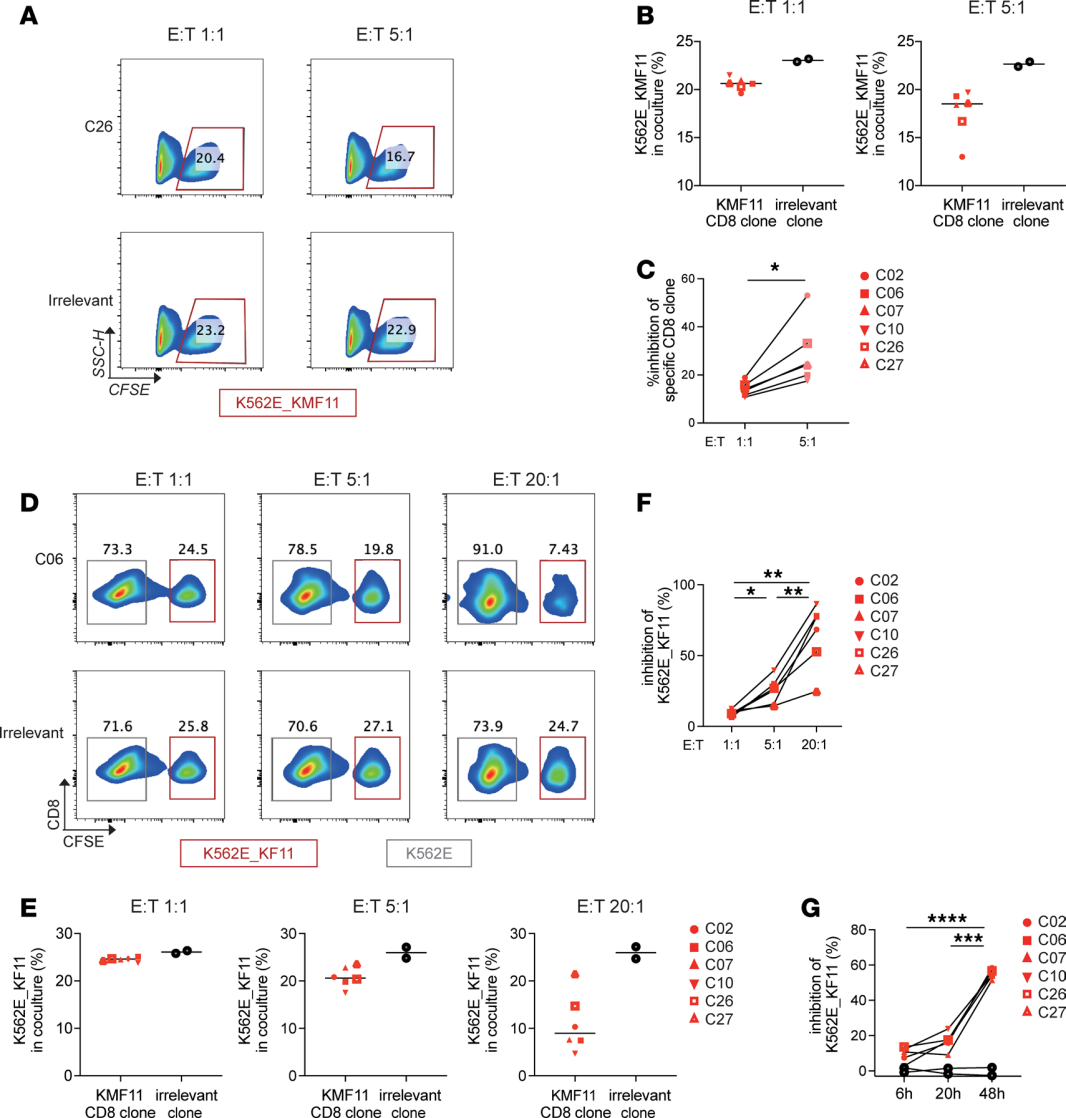
KMF11-specific HLA-E–restricted CD8^+^ T cells lyse antigen-expressing K562E cells. (**A**) K562E_KMF11 SCT cells (CellTrace CFSE-labeled) were mixed with control K562E cells and K562E_Mtb44 SCT–expressing cells at 1:1:1. These cells were cocultured with cloned KMF11-specific HLA-E–restricted CD8^+^ T cells for 48 hours at E/T ratios as shown, in duplicate. Flow plots of clone C26 and a control clone are shown, gating on viable CD3^–^CD8^–^ cells, quantifying K562E_KMF11 target cells remaining, boxed in red. (**B**) Percentage of CFSE-positive K562E_KMF11 SCT cells after coculture with KMF11-specific CD8^+^ T cell clones (red symbols) or 2 irrelevant ECOV2 CD8^+^ T cell clones (gray dots) at E/T 1:1 and 5:1. (**C**) Reduction of KMF11 SCT–expressing cells by KMF11-specific, HLA-E–restricted CD8^+^ T cells, using the formula [1 – (% target cells with specific T clone/% target cells with irrelevant control T clone)] × 100 at E/T ratio of 5:1 compared with 1:1. Significance assessed using Wilcoxon’s signed-rank test. *P < 0.05. Each clone is denoted using different symbols. Two independent experiments were performed. (**D**) CellTrace CFSE–labeled K562E_KF11 SCT cells were mixed with K562E cells and then cocultured with KMF11-specific HLA-E–restricted CD8^+^ T cells for 48 hours, in duplicate, at E/T ratios shown. The percentage of K562_KF11 cells remaining is boxed in red. (**E**) The percentages of CFSE-positive K562E_KF11 SCT–expressing cells after coculture with KMF11-specific CD8^+^ T cell clones (red symbols) or 2 irrelevant ECOV2 CD8^+^ T cell clones (gray dots). (**F**) Increasing E/T ratios of 1:1 to 5:1 and 20:1 enhanced lysis of K562E-KF11 cells. Significance determined by 1-way ANOVA with Tukey’s multiple-comparison test. *P < 0.05, **P < 0.01. (**G**) Time-dependent inhibition of K562E_KF11–expressing cells by CD8^+^ T cell clones. Two irrelevant T cell clones were negative controls. Significance tested by 1-way ANOVA with Tukey’s multiple-comparison test. ***P = 0.0001, ****P < 0.0001. Two independent experiments were performed.

We then sought to determine whether the CD8^+^ T cell clones could eliminate target K562E cells expressing KMF11, by establishing a flow-based assay where CD8^+^ T cell clones were cocultured with K562E_SCT–expressing target cells. In these cocultures, the target K562E_KMF11 SCT–expressing cells or the wild-type KF11 SCT–expressing target K562E_KF11 cells were prelabeled with CellTrace CFSE and subsequently mixed with both K562E_Mtb44 SCT–expressing cells (as a specificity control) and K562E (background control) cells at 1:1:1 ratio. If the clone is peptide specific, there should be fewer target cells remaining after coculture compared with K562E_Mtb44 SCT and K562E cells when compared with numbers of target cells in the incubations with the irrelevant CD8 clone control. Cocultures were conducted at effector-to-target (E/T) ratios of 1:1 and 5:1 (and 20:1 in the K562E_KF11 SCT/CD8 cocultures) using the previously identified 6 responsive CD8^+^ T cell clones (C02, C06, C07, C10, C26, C27) with 2 irrelevant ECOV2 CD8^+^ T cell clones as controls. After a 48-hour coculture period, the cells were collected, and flow cytometry was performed to gate viable CD3, CD8, and double-negative cells (K562E cells) for further quantification of the remaining percentage of K562E_KMF11 SCT– or KF11 SCT–expressing target cells (CFSE positive) (Supplemental Figure 3; shown for clone C26 in Figure 4A and clone C06 in Figure 4D, respectively). The inhibitory effect of KMF11-specific HLA-E–restricted CD8^+^ T cells, calculated as [1 – (% target cells with specific T clone/% target cells with irrelevant control T clone)] × 100], was used to test the specific reduction of target cells.

Initial coculture experiments using K562E_KMF11 SCT target cells revealed a modest reduction in target cells in the presence of KMF11-specific CD8^+^ T cell clones at both the E/T ratios of 1:1 and 5:1, while irrelevant ECOV2 CD8^+^ T cells showed minimal effect on target cell survival (Figure 4B). The reduction was dependent on a higher E/T ratio (*P < 0.05, Wilcoxon’s signed-rank test; Figure 4C). We next investigated whether KMF11-primed CD8^+^ T cell clones could cross-recognize and inhibit target cells expressing the WT KF11 peptide. In the coculture assay, we used K562E_KF11 SCT–expressing target cells at E/T ratios of 1:1, 5:1, and 20:1 (Supplemental Figure 3 and Figure 4D). After the 48-hour coculture, KMF11-specific CD8^+^ T cell clones mediated a modest reduction of K562E_KF11 SCT–positive target cells at an E/T 1:1 ratio (Figure 4E). The effect was enhanced at higher E/T ratios, with target cell percentages dropping to 8.9% (range: 4.7%–21.5%) at an E/T ratio of 20:1 (Figure 4E). Remarkably, 6 specific CD8^+^ T cell clones (C10, C06, C02, C26, C07, C27) exhibited particularly potent and significant incremental reduction of K562E_KF11 SCT–positive cells across all 3 ratios (*P < 0.05, **P < 0.01, 1-way ANOVA test followed by Tukey’s multiple-comparison test; Figure 4F). A subsequent time course assay performed at an E/T ratio of 20:1 for T cell clones C02, C06, C07, C10, and C26 with 2 irrelevant CD8^+^ T cell clones as negative controls demonstrated the progressive reduction of K562E_KF11 SCT–expressing cells by 5 KMF11-specific CD8^+^ T cell clones when tracked over time points of 6, 20, and 48 hours after coculture. This progressive pattern confirmed time-dependent inhibition (***P = 0.0001, ****P < 0.0001, 1-way ANOVA with Tukey’s multiple-comparison test; Figure 4G). Together, the data indicate that KMF11-specific HLA-E–restricted CD8^+^ T cells could eliminate target cells presenting the WT KF11 antigen as an SCT in a dose-dependent and time-dependent manner, also suggesting that relatively high E/T ratios and longer time interactions may be important.

### Antiviral effect of KMF11-specific TCR-transduced primary CD8^+^ T cells.

**Figure 5:**
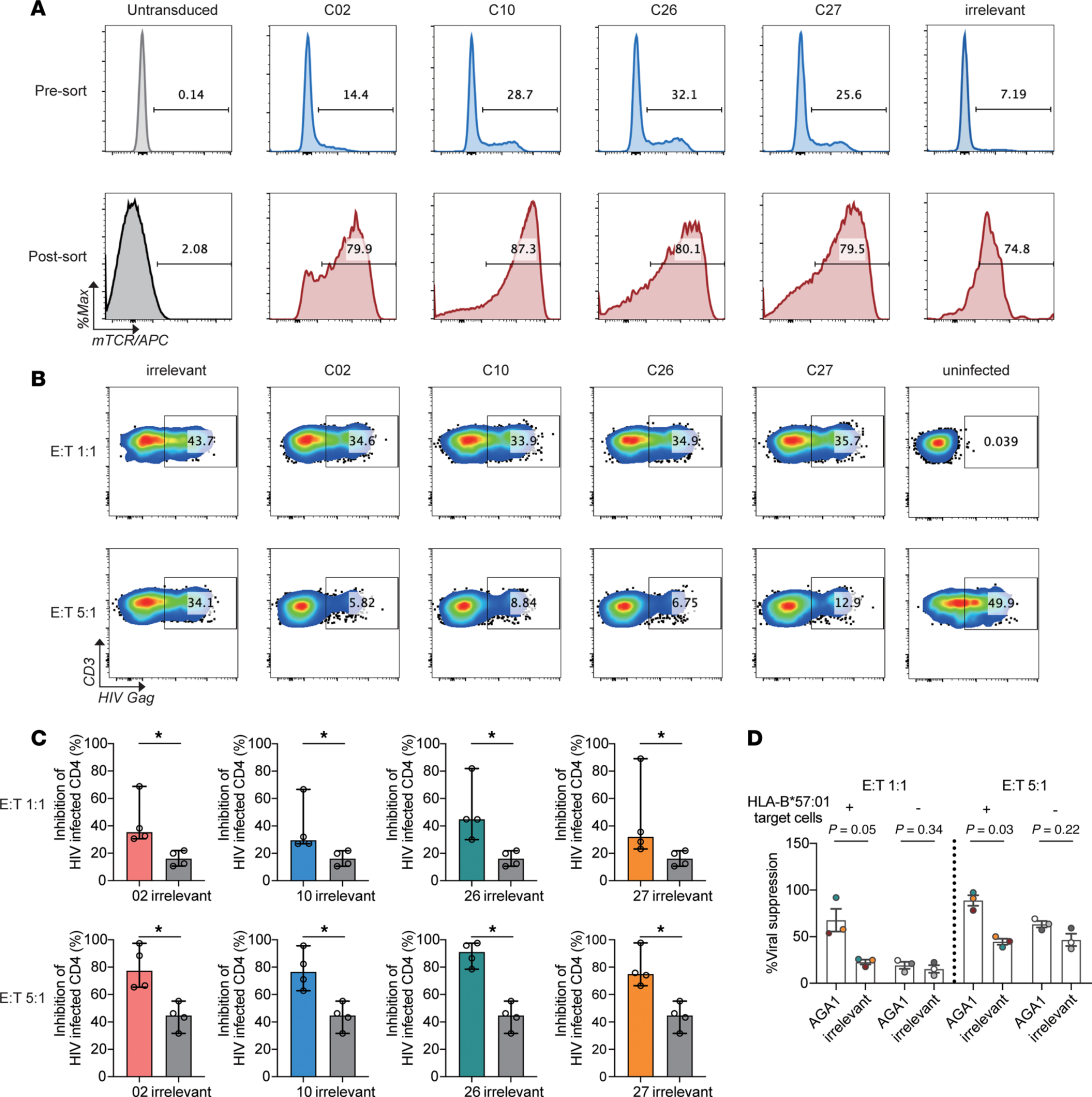
Antiviral effect of KMF11-specific TCR CD8^+^ T cell transductants against HIV-infected primary CD4^+^ T cells in a viral suppression assay. (**A**) KMF11-specific TCRs (C02, C10, C26, C27) and an irrelevant HLA-E–restricted TCR specific for a SARS-CoV-2–derived peptide were transduced into primary CD8^+^ T cells and then stained with anti-mouse Cβ antibody, anti-CD8/CD3, and Live/Dead Fixable Aqua Dead Cell Stain kit. Percentages of mTCR^+^ cells are indicated. Postenrichment staining of mTCR^+^CD8^+^ T cell transductants for each TCR is shown. Untransduced CD8^+^ T cells were included as controls. (**B**) CD8^+^ T cell transductants were cocultured with HIV-infected primary CD4^+^ T cells at E/T ratios of 1:1 and 5:1 for 5 days. Intracellular HIV Gag staining (KC57-FITC) was performed after surface staining with anti-CD8/CD3/CD4 and Live/Dead Fixable Aqua Dead Cell Stain kit. The percentages of HIV Gag expression in HIV-infected primary CD3^+^CD8^–^ T cells were assessed. Uninfected CD4^+^ T cells and HIV-infected CD4^+^ T cells without effectors were included as negative and positive controls, respectively. (**C**) The suppressive effect of KMF11-specific TCR transductants against HIV-infected CD4^+^ T cells was assessed at E/T ratios of 1:1 and 5:1. Data are represented as median with 95% CI from 2 independent experiments (*n* = 4 donors). Statistical significance between KMF11 TCR transductants and irrelevant TCR controls was determined using the Mann-Whitney *U* test (*P < 0.05). (**D**) Suppression of HIV-infected CD4^+^ T cells by HLA-B*57:01–restricted TCR transductants (AGA1) was evaluated at E/T ratios of 1:1 and 5:1. Data are shown as mean ± SEM from 2 independent experiments. CD4^+^ T cell targets were derived from 6 donors, comprising 3 HLA-B*57:01–positive and 3 HLA-B*57:01–negative individuals. Each donor is represented by a distinct colored circle. Statistical comparisons between AGA1 TCR transductants and irrelevant TCR controls were performed using a 2-tailed paired *t* test.

To assess whether the KMF11-specific TCRs confer antiviral recognition, we transduced TCRs into fresh CD8^+^ T cells. We chose the TCRα and -β chain sequences from 4 CD8^+^ T cell clones, C02, C10, C26, and C27, fused onto murine TCR constant regions (mTCRs). These products were introduced into lentiviral constructs and subsequently transduced into activated primary CD8^+^ T cells from healthy donors according to our established method (7). The TCRs transduced were KMF11-specific TCRs from clones C02, C10, C26, and C27 or an irrelevant HLA-E–restricted TCR specific for SARS-CoV-2 (1016 clone 1), which had demonstrated a suppressive effect on infected Calu-3 cells in our previous study (7). As a further positive control, we generated lentiviral expression constructs for the HLA-B*57:01–restricted KF11-specific TCRs from the Aga-a clone (21, 22) and also transduced this into fresh, activated CD8^+^ T cells. We demonstrated successful enrichment of mTCR-positive CD8^+^ T cells by FACS analysis, finding similar levels of TCR expression for each of the TCRs (Figure 5A).

We next questioned the capacity of primary CD8^+^ T cells transduced with these TCRs to suppress HIV-1 replication in vitro. Replication-competent VSV-G pseudotyped HIV NL4-3 virus was used to infect previously activated, primary CD4^+^ T cells from healthy donors. KMF11-specific or control TCR CD8^+^ T cell transductants were then cocultured with these HIV-infected primary CD4^+^ T cells for 5 days at E/T ratios of 1:1 and 5:1. Using flow cytometry, the proportion of HIV-1 Gag-positive cells in the viable CD3^+^CD8^–^ T cell population was gated and analyzed to reveal the suppressive effect at both E/T ratios for the TCRs (Supplemental Figure 4 and Figure 5B). Notably, 4 KMF11-HLA-E–specific TCR transductants (C02, C10, C26, C27) mediated significantly greater suppression of HIV-infected CD4^+^ T cells when compared with the irrelevant, SARS-CoV-2–specific TCR control (Figure 5C). At an E/T ratio of 1:1, the median suppression mediated by these transductants ranged from 30% to 45% compared with 16% for the irrelevant controls. This enhanced suppression was more pronounced at an E/T ratio of 5:1, with medians of 75% to 91% versus 45% for the controls (Figure 5C). The relatively high backgrounds with the control SARS-CoV-2–specific TCR-transduced CD8^+^ T cells likely reflected cytokine production resulting from their activation and expansion, likely causing nonspecific killing of HIV-infected cells.

To benchmark the findings, we evaluated a canonical HLA-B*57:01–restricted TCR, AGA1, that we previously reported (27, 28). Using pseudo-typed HIV NL4-3–infected CD4^+^ T cells from 3 HLA-B*57:01–positive donors and 3 negative donors, we confirmed that AGA1 specifically targeted HLA-matched cells. This TCR exhibited significant antiviral activity against HLA-B*57:01–positive targets at E/T ratios of 1:1 and 5:1, despite some background at E/T 5:1, while showing no effect against HLA-B*57:01–negative cells, thereby confirming HLA restriction (Figure 5D). The 4 HLA-E–restricted TCR transductants consistently suppressed target cells from all 6 donors (Supplemental Figure 5), confirming the reproducibility of the viral suppression assay. The data illustrate the difference between classically HLA-restricted T cells and the universality of HLA-E–restricted T cells (Figure 5C). Collectively, the result indicates that KMF11-stimulated, KF11-reactive TCR CD8 transductants efficiently suppress HIV-infected primary CD4^+^ T cells in a dose-dependent manner.

## Discussion

A comprehensive understanding of the priming mechanisms and functional roles of unconventional HLA-E–restricted CD8^+^ T cells in HIV infection is crucial for developing immunotherapies and vaccine strategies. Having previously identified 2 HIV-derived HLA-E–restricted epitopes (18, 19), we set about investigating the antiviral activity of the recently described HLA-E–restricted HIV Gag_162-172_ (KAFSPEVIPMF, KF11) epitope (20). This natural B*57:01-restricted epitope is one of a number of Gag-derived epitopes associated with sustained long-term nonprogression in people living with HIV. Bansal et al. (20) found KF11 epitope–specific HLA-E–restricted responses in vivo in people with HIV who were positive for HLA-B*57:01. However, in their study, HLA-E tetramers were not generated due to the instability of KF11 in complex with HLA-E. In our HLA-E binding assays where the peptide is added to peptide-free HLA-E–β2m complexes, KF11 bound very weakly to HLA-E (~10% of the level of VL9 when tested by sandwich ELISA (25), a level of binding that does not enable production of stable HLA-E tetramers (18). In humans, HLA-E predominantly binds the canonical HLA-class I signal peptide VMAPRT(V/L) (L/V/I/F)L (VL9), but can also bind pathogen-derived peptides, including strong binders such as VMAPRTLIL from HCMV UL40 (29), RLPAKAPLL from *Mycobacteria tuberculosis* (3), VMPLSAPTL from SARS-CoV-2 NSP13 (7), and more modest binders Mtb14 (RMAATAQVL) from *M*. *tuberculosis* (30) and RL9HIV (RMYSPTSIL) from HIV (24). All of these peptides have leucine or methionine at position 2 of the peptide, with the side chain anchored in the B pocket of HLA-E. We found that substituting methionine for alanine at position 2 in KF11 — giving the peptide termed KMF11 — dramatically enhanced binding to HLA-E. This was shown by nano-DSF measured T_m_, which increased from 43°C to 51°C and separately by direct binding in a peptide-exchange ELISA (Figure 1). Thus, modifying the key anchor residue at position 2 stabilized peptide binding and facilitated successful tetramer generation. Although similar strategies have been explored for MHC class I, such as C-trap linkage (18) or homo-homo cysteine modifications (31), our approach offers a more natural alternative that likely minimizes structural alterations to the HLA-E–peptide interface.

The KMF11/HLA-E tetramers facilitated the isolation and growth of KMF11-specific T cell clones from in vitro KMF11 peptide–primed PBMCs from HLA-B*57:01–negative donors. Functional analyses revealed that a subset of these clones exhibited cross-reactivity with the native KF11 peptide when assessed using genetically modified K562 cells that expressed the peptide bound to HLA-E in an SCT format, as antigen-presenting cells. Crucially, 4 TCRs were identified with the ability to suppress HIV-1 NL4-3 replication in primary CD4^+^ T cell cultures in vitro, indicating that this weak binding epitope is naturally presented by HLA-E during viral infection.

Since we confirmed that KF11 exhibits very weak binding to HLA-E, consistent with the findings of Bansal et al. (20), it seems likely that induction of KF11-specific HLA-E–restricted CD8^+^ T cell responses arise in people with HIV who are positive for HLA-B*57:01 because of cross-reactivity mediated by T cells primed by HLA-B*57:01-KF11. Disease control in HIV infection has often been associated with distinct features of TCR clonotypes, including broader cross-reactivity to variant viral peptides and enhanced functional capabilities (32, 33). For HLA-B*57:01–restricted KF11 T cells, we and others have previously identified an immunodominant Vα5/Vβ19 TCR (AGA1) in people with HIV who are positive for HLA-B*57:01, which sees this conserved KF11 peptide and also cross-recognizes its rarer variants (27, 28, 34, 35). Furthermore, using a yeast display library, we recently demonstrated that this same TCR can cross-react with microbe-derived peptides that only in part resemble the KF11 sequence (36), illustrating the cross-reactivities of TCRs and highlighting a potential role for microbial antigens in shaping specific T cell immunity, and perhaps the frequency of Vα5/Vβ19 usage. Thus, the initial immune response in HLA-B*57:01–positive individuals may primarily be directed against HLA-B*57:01–restricted KF11, with a subset of these T cell clones showing cross-reactivity to KF11 via HLA-E restriction. The rarity of naturally occurring KF11-specific T cell responses in people with HIV who lack HLA-B*57:01 could reflect the more stringent requirements for T cell priming compared with targeting. Although enough KF11 binds to HLA-E to make a cell recognizable by T cells, as shown here in the HIV virus suppression assay, there may be insufficient surface expression to prime T cells in vivo. The cross-reaction hypothesis aligns with Bansal et al.’s observation of a shared group of β chain TCR usage between HLA-B*57– and HLA-E–restricted responses. A short sequence match was also seen here within the α chain CDR3s of clone 26 and the HLA-B*5701-KF11–specific Aga-1 TCR (21).

It is possible that very low-affinity TCRs that are close to, or even just below, the threshold of tetramer staining could still be functionally active in vivo. This phenomenon has been observed in cases where multimer staining fails to detect self-targeted T cell responses in cancer or autoimmune diseases (37–43), as well as in MHC-II–restricted antigen-specific CD4^+^ T responses (44–46). In our study, HLA-E–restricted CD8^+^ T cell clones exhibited only partial staining with HLA-E tetramers, a pattern also observed in our previous HIV-1 and COVID-19 studies (7, 18), as well as in other studies involving pathogen-specific or tumor-specific TCRs (7, 18, 47, 48). These findings are likely caused by the low affinity of TCRs and/or low density of TCR surface expression induced during long-term coculture (49). Detection methods with higher-order multimers (e.g., spheromers, dextramers) (42, 50) might improve staining.

In our in vitro recognition and coculture assays, the HLA-E–restricted KF11-specific clones demonstrated only a modest ability to secrete cytokines and to express activation markers upon antigen stimulation in commonly used short-term assays. This has been observed previously for other HLA-E–restricted T cell responses (7, 18) and likely reflects the cumulative effect of low-affinity interactions between peptide-HLA-E and then with the TCRs. The latter is evidenced both by tetramer staining and the delayed viral suppression kinetics observed here, compared with what is typically observed for higher-affinity classical HLA-Ia–restricted TCRs. This aligns with findings demonstrating that CD8^+^ T cells can mount detectable responses to low-affinity antigens (51). Similarly, low-affinity TCR interactions in tumor microenvironments have been shown to effectively mediate antitumor responses while preventing excessive T cell activation and subsequent exhaustion (52). As an index of T cell priming and activation, T cell responses may also be influenced by additional factors, such as co-receptor (CD8 or CD4) involvement and regulatory (stimulatory/inhibitory/adhesion) molecule stimulation. For example, Denkberg et al. found anti-CD8 antibodies could block the tetramer binding of a human tumor-specific CD8 clone, indicating the important role of CD8 in pHLA-TCR interactions (53). Despite the suboptimal functional profile, the clones with cytotoxicity exhibited a suppressive effect against KF11-expressing target cells over 48 hours, demonstrating their functional capacity for antigen-specific killing. Importantly, CD8^+^ T cells transduced with TCRs from 4 of these clones also inhibited replication of HIV-1 NL4-3, which expresses only WT KF11, in primary CD4^+^ T cells in vitro. The CD4^+^ T cell donors were both positive and negative for HLA-B*57:01. In contrast, the classical HLA-B*57:01–restricted TCR AGA1 only showed a suppressive effect on HIV-infected CD4 cells from HLA-B*57:01–positive donors. Thus, HLA-E–restricted T cells could contribute to viral control in donors lacking HLA-B*57:01.

HLA-E is monomorphic in its peptide-binding cleft, giving it a potential advantage over highly polymorphic classical HLA-Ia molecules in vaccine design and other immunotherapeutic interventions. This is particularly important in the context of HIV-1 and other viral infections or tumor environments, where HLA-Ia molecules are often significantly downregulated to evade classical CD8^+^ T cell responses, while HLA-E expression is typically upregulated, or remains undisrupted, to evade NK cell attack. In the context of cancer immunotherapy, there has also been recent interest in the role of low-avidity T cells, which have been shown to contribute to tumor control, while their high-avidity counterparts can become exhausted and lose cytotoxic functionality (47, 54). Collectively, these findings indicate that with low avidity, functionally suppressive HLA-E–restricted T cells might provide an alternative or complementary strategy to conventional HLA-Ia–restricted immune responses to broaden the efficacy of T cell–based therapies.

A primary limitation of our study is the lack of resolved crystal structures for the pHLA-E/TCR complex, which could reveal details of specific molecular interactions between the KF11/HLA-E complex and TCRs. Although we have shown KMF11-specific TCRs can effectively suppress WT HIV-1 NL4-3 replication through KF11 recognition, future studies using epitope-mutant viruses and comprehensive peptide screening would be valuable to address whether there is cross-reactivity with other HIV peptides or the potential for mutant escape. Additionally, validation of these findings in vivo will be required. Such validation would be important to assess the killing capacity of these TCRs, particularly in tissue compartments where HLA-E is enriched and may be upregulated, and where classical HLA-Ia molecules are downregulated by the viral infection. Such models (55) also offer the advantage of conducting the experiments over extended time periods, in contrast with the commonly used short-term in vitro assays of T cell function.

In summary, our findings demonstrate that peptide modifications can enable priming of cross-reactive, functionally effective HLA-E–restricted T cells. This study extends the avenue toward HLA-E–centric vaccines and immunotherapies, which may circumvent viral HLA-Ia downregulation as an immune evasion mechanism while leveraging the broad global conservation of HLA-E, offering a strategy for treating persistent infections cause by HIV and other viruses.

## Methods

### Sex as a biological variable.

Our study used PBMCs obtained from leukapheresis cones from NHS Blood and Transplant, UK, and included both sexes. Sex was not considered as a biological variable.

### Cell lines and primary cells.

The MHC-I–null K562 cell line was used as the antigen-presenting cells or target cells in the antigen recognition assay and coculture assays (18). These cells were genetically modified to express the HLA-E SCT, which incorporates peptide, HLA-E heavy chain, and β2m. The K562 cells transfected with HLA-E*01:03 (K562E) were provided by Thorbald van Hall (Leiden University Medical Center, Netherlands) (56). The K562 cell line was obtained from the European Collection of Authenticated Cell Cultures (ECACC). HEK293T cells were obtained from the ECACC and were used for producing the lentivirus for cell transduction for TCRs and to generate HLA-E SCT constructs. PBMCs from healthy male and female donors were isolated from leukapheresis cones obtained from NHS Blood and Transplant, UK, and HLA-typed PBMCs were obtained from Cambridge Bioscience Ltd. Both sources obtained ethical approval and informed consent from the volunteer donors. Received blood cells were handled under the UK Human Tissue Authority requirements. PBMCs were used to generate T cell clones and TCR transductants, to make CD4^+^ T cells for HIV-1 infection in vitro, and for the supply of irradiated mixed lymphocyte feeder cells to expand T cell clones or transductants. CD4^+^ or CD8^+^ T cells were positively selected from PBMCs using magnetic beads according to the manufacturer’s instructions.

### Peptides.

HLA-B leader sequence peptide VMAPRTVLL (VL9) and candidate peptides including KF11 (KAFSPEVIPMF), KMF11 (KMFSPEVIPMF), Mtb44 (RLPAKAPLL, and RL9HIV (RMYSPTSIL) were synthesized by Genscript (>90% purity). A UV-labile HLA-B leader-based peptide (VMAPRTLVL) incorporating a 3-amino-3-(2-nitrophenyl)-propionic acid residue substitution at position 5 (J residue) was synthesized by Dris Elatmioui at Leiden University Medical Center, Leiden, Netherlands. Lyophilized peptides were initially reconstituted to 200 mM or 100 mM in DMSO and aliquoted for cryopreservation at –80°C until further use.

### Protein expression, purification, and refolding.

The details of HLA-E heavy-chain expression, including inclusion body preparation, solubilization, refolding, and final purification, were performed according to protocols that have been previously described (7, 16, 18, 24). Briefly, HLA-E*01:03 heavy chain and β2m were cloned into expression vector, expressed in BL21 DE3pLysS-competent *E*. *coli* cells, and purified as inclusion bodies. After solubilization in urea buffer, HLA-E*01:03 protein was refolded in Tris-arginine/glutathione redox buffer with peptide. Complexes were filtered, concentrated, and purified by Superdex S75 16/60 chromatography. The protein was then aliquoted for further analysis by SDS-PAGE to confirm the presence of HLA-E heavy chain and β2m.

### DSF.

The thermal stability of no-peptide and peptide-loaded HLA-E was determined by DSF using Prometheus Panta instrumentation (Nanotemper). In brief, 0.45 μg/μL of HLA-E was incubated with 10 M excess peptide in a 20 μL final volume of 50 mM Tris pH 7, 150 mM NaCl buffer for 30 minutes. After incubation, approximately 20 μL of individual samples were split between 2 Prometheus Panta nanoDSF Grade Standard Capillaries (Nanotemper) and transferred into a capillary sample holder. Excitation power was pre-adjusted to obtain a range between 8,000 and 15,000 raw fluorescence units for fluorescence emission detection at 330 nm and 350 nm. A thermal ramp of 1°C/min from 20°C to 95°C was applied. Thermal melt data calling was automatically generated using PR.Panta Analysis software (v1.2).

### Generation of antigen-presenting cells.

SCT constructs incorporating HLA-E *01:03 heavy chain, β2m light chain, and different peptides (KF11, KMF11, ECOV2P1, and Mtb44) were genetically modified and confirmed as previously described. The lentivirus was generated with the above SCT constructs, and then transduced into K562 cell lines to generate antigen-presenting cells with different peptides for further functional assays (K562E_KF11, K562E_KMF11, K562E_ECOV2, and K562E_Mtb44).

### HLA-E binding peptide-exchange ELISA.

A highly sensitive HLA-E binding ELISA was conducted as previously described (18, 25). Briefly, refolded HLA-E proteins (0.5 μM) preloaded with a labile VL9 variant peptide (7MT2) were incubated overnight with excess tested peptides (100 μM) in a reaction buffer containing 400 mM L-arginine monohydrochloride, 100 mM Tris, 5 mM reduced glutathione, 0.5 mM oxidized glutathione, and 2 mM EDTA. The reaction mixture was then diluted 1:100 in PBS containing 2% BSA, and 50 μL was added to ELISA plates precoated with 20 μg/mL anti-human HLA-E monoclonal antibody (3D12, BioLegend). After 1 hour of incubation, the plates were washed with PBS containing 0.05% Tween-20 and treated with 2 μg/mL anti-human β2m HRP-conjugated IgG antibodies (PA1-29662, Invitrogen) for 30 minutes. After additional wash steps, 50 μL of enhancement reagent (Dako EnVision, diluted in PBS/2% BSA with 1% normal mouse serum) was added to amplify the HRP signal. Subsequently, 100 μL of TMB substrate was applied for development, and the reaction was stopped using 100 μL of STOP Solution. Absorbance was measured at 450 nm using a FLUOstar OMEGA reader. Each peptide was tested in 3 independent peptide-exchange reactions, with duplicates from each reaction analyzed. We used a VL9-positive control and a peptide-free no-rescue control to normalize background and calculate binding affinity as a percentage of VL9 binding. HLA-E binding rankings were determined using the formula (average signal of the tested peptide − average DMSO signal)/(average VL9 signal − average DMSO signal).

### HLA-E tetramer generation and staining on CD8^+^ T cells.

Biotinylated HLA-E*01:03 monomers subjected to UV peptide exchange were conjugated to streptavidin-bound phycoerythrin or allophycocyanin at a molar ratio of 4:1, following a previously described protocol (18). Additionally, conventional tetramers were prepared for VL9 (VMAPRTVLL). KMF11-primed CD8^+^ T cells or CD8^+^ T cell clones were stained with UV-exchanged KMF11/HLA-E tetramers (0.5 μg per 1 × 10^6^ cells) for 45 minutes at room temperature in the dark. After staining, cells were washed with PBS and subsequently labeled with Live/Dead Fixable Aqua dye and flow cytometry antibodies, including anti-CD3-APC-Cy7 (300318, BioLegend), anti-CD4-PerCP-Cy5.5 (344607, BioLegend), anti-CD8-BV421 (301036, BioLegend), anti-CD94-FITC (305504, BioLegend), and anti-CD56-BV510 (362534, BioLegend), for 20 minutes at room temperature in the dark. After staining, cells were washed, fixed in 2% paraformaldehyde, and acquired using an LSRFortessa cytometer (BD Biosciences) or Attune NxT flow cytometer with CytKick Max (Thermo Fisher Scientific). Data were analyzed using FlowJo software v10.10.0 (Tree Star).

### Induction of HLA-E–restricted CD8 response using DC differentiation T cell–priming assay.

PBMCs from 2 healthy donors were primed to induce the HLA-E–restricted CD8^+^ T cell response using a modified DC differentiation T cell–priming protocol reported previously. First, PBMCs were cultured at 1 × 10^7^/mL in 6-well plates with KMF11 peptide (50 μM) added on day 1 in AIM-V medium supplemented with GM-CSF and IL-4. A cocktail of cytokines (IL-1β, TNF-α, prostaglandin E_2_, IL-7, and IL-15) were supplemented to differentiate and mature DCs and expand T cells at day 1. On day 6, cells were collected and transferred into fresh complete media with IL-2, IL-7, and IL-15 to support T cell homeostasis. The flow staining with HLA-E tetramers and flow antibodies to surface markers was performed on day 8.

### Live-cell sorting and generation of HLA-E tetramer–positive CD8^+^ T cell clones.

KMF11-primed CD8^+^ T cells from healthy donors were first stained with KMF11/HLA-E tetramers conjugated to allophycocyanin and phycoerythrin for 45 minutes at room temperature in the dark. After a PBS wash, cells were stained for 20 minutes at room temperature in the dark using Live/Dead Fixable Aqua, anti-CD3-APC-Cy7 (300318, BioLegend), anti-CD4-PerCP-Cy5.5 (344607, BioLegend), anti-CD8-BV421 (301036, BioLegend), anti-CD94-FITC (305504, BioLegend), and anti-CD56-BV510 (362534, BioLegend). CD3^+^CD4^−^CD56^−^CD94^−^CD8^+^Tetramer^+^ T cells were live-sorted using a FACSAria III sorter (BD Biosciences). Sorted cells were subsequently seeded into 384-well plates at a density of 0.4 cells per well, along with irradiated (45 Gy) allogeneic feeder cells (from 3 healthy donors, 2 × 10^6^ cells/mL), and stimulated with PHA (1 μg/mL) and IL-2 (500 U/mL) in complete media. The complete media consisted of RPMI 1640, 10% AB human serum (UK National Blood Service), 1% penicillin/streptomycin, 1% glutamine, 1% sodium pyruvate, 1% nonessential amino acids, and 0.1% β-mercaptoethanol. After 12 days, T cell clones were further expanded using irradiated feeder cells with PHA and IL-2. Tetramer positivity was tested on expanded CD8^+^ T cell clones to confirm their specificity. Functional assessments of CD8^+^ T cell clones were then conducted as described in subsequent sections.

### TCR sequencing of HLA-E–restricted CD8^+^ T cell clones.

RNA was extracted from CD8^+^ T cell clones using the RNeasy Plus Mini Kit (QIAGEN). Approximately 100 ng of RNA was used to generate TCR libraries with the SMARTer Human TCR α/β Profiling Kit v2 (Takara Bio), following the manufacturer’s protocol, which leverages SMART and 5′ RACE technologies. Full-length TCRα and -β chain sequences were obtained using a MiSeq Reagent kit v3 (600-cycle) on an Illumina MiSeq platform. The raw BCL files were converted to FASTQ format using bcl2fastq (v2.20.0.422). Cogent NGS Immune Profiler (v2.0, Takara Bio) was used to analyze the profile of full-length TCRα and -β chain sequences.

### Functional assessment of KMF11-specific HLA-E–restricted CD8^+^ T cells.

To assess the functionalities of CD8^+^ T cell clones, cells were rested in complete medium overnight before being cocultured with genetically modified K562 cell lines (null of classical HLA-I expression) including KMF11, KF11, or ECOV2P1 expressing HLA-E SCT–transduced K562 cell lines. HLA-E–expressing K562 cells were used as reference control. The coculture was conducted at a CD8/K562 ratio of 1:1 for 10 hours. For maximal functionality assessment, CD8^+^ T cell clones were also treated independently with PMA/Ionomycin. Brefeldin A (5 μg/mL) and GolgiStop (5 μg/mL) were added after 1 hour of incubation, and anti-CD107a-BV421 and anti-CD107b-BV421 were included at the start of the coculture. After incubation, cells were stained with Live/Dead Fixable Aqua and surface marker antibodies (anti-human CD3 and anti-human CD8) in PBS. Cells were then fixed and permeabilized using Cytofix/Cytoperm (BD Biosciences). Intracellular staining was performed using fluorochrome-conjugated antibodies targeting TNF-α (502909, BioLegend), IFN-γ (502528, BioLegend), CD137 (309810, BioLegend), PD-1 (335714, BioLegend), and CTLA-4 (369610, BioLegend) in perm/wash solution. Data acquisition was carried out on an Attune NxT flow cytometer with CytKick Max (Thermo Fisher Scientific), and results were analyzed using FlowJo v10.10.0 (Tree Star).

### Inhibition effect of KMF11-specific HLA-E–restricted CD8^+^ T clones assessed using coculture assay.

CellTrace CFSE Cell Proliferation Kit (Invitrogen) prepared at a concentration of 0.8 μM in prewarmed PBS was used to trace the target cells. Next, 5 × 10^6^ to 10 × 10^6^ K562E_KMF11 (or K562E_KF11) cells were washed with 10 mL PBS and then labeled with prewarmed CellTrace CFSE at 100 μL per 1 × 10^6^ cells for 20 minutes at 37°C in the dark. Complete media was then added for 5 minutes to quench the reaction. After centrifugation at 350*g* for 5 minutes, the cells were resuspended and mixed with HLA-E expressing K562 (K562E) cells at the ratio of 1:1 and plated at 0.5 × 10^5^ cells per well in duplicates in 96-well plates. The concentration of CD8^+^ T cell clones or transductants was washed and adjusted to coculture with K562 target cells at different E/T ratios of 1:1, 5:1, or 20:1 at 37°C in a 5% CO_2_ incubator for 48 hours. The samples were washed with PBS and then stained with Live/Dead Fixable Aqua, anti-CD3-APC-Cy7 (300318, BioLegend), and anti-CD8-BV421 (301036, BioLegend) for 15 minutes at room temperature. The cells were subsequently washed with PBS and fixed with 2% PFA for sample acquisition on the Attune NxT flow cytometer (Thermo Fisher Scientific). Data were analyzed using FlowJo software v10 (Tree Star). To assess the suppressive effect of CD8 cells, viable cells that were negative for both CD3 and CD8 were gated to further assess the percentage of remaining CFSE-positive K562E_KMF11 (or K562E_KF11) target cells. Percentages of inhibition of K562E_KMF11 (or K562E_KF11) target cells were calculated as (percentage of CFSE-positive cells in irrelevant clone control – percentage of CFSE-positive cells in KMF11- or KF11-specific CD8^+^ T cell clone condition)/percentage of CFSE-positive cells in irrelevant clone control) × 100%.

### TCR transduction into primary CD8^+^ T cells.

Primary CD8^+^ TCR transductants were generated as previously described (7). Briefly, TCRα and -β VDJ regions were amplified and assembled into a pHR-SIN backbone with the murine TCRα and -β constant regions using the HiFi DNA Assembly cloning kit (New England Biolabs). Lentiviruses encoding HLA-E–restricted TCRs were generated by transfecting HEK293T cells with packaging plasmids pMD.G, pCMV-dR8.91, and pHR-SIN-TCR using Turbofectin (Origene). Primary CD8^+^ T cells were isolated from PBMCs using CD8 MicroBeads (Miltenyi Biotec) and activated with CD3/CD28 Dynabeads (Thermo Fisher Scientific) in T cell medium supplemented with IL-2 and IL-15. Activated CD8^+^ T cells were then transduced with freshly filtered lentiviruses. Transduction efficiency was evaluated on day 7 by flow cytometry using anti-mouse TCR-β antibody (clone H57-597, BioLegend). TCR-β^+^CD8^+^ T cells were isolated by cell sorting (BD Fusion) and expanded for a further 10–12 days before the downstream functional assessment of TCR-transduced T cells.

### Viral suppression assay.

PBMCs were freshly separated from healthy donors, and CD4^+^ T cells were isolated from PBMCs using positive selection with anti-human CD4 magnetic beads, following the manufacturer’s protocol (MACS, Miltenyi Biotec). CD4^+^ T cells were then activated with anti-human CD3 monoclonal antibody at the concentration of 100 ng/mL (clone OKT3, Invitrogen) in RPMI 1640 complete medium supplemented with 5% AB human serum, 1% penicillin/streptomycin, and IL-2 (50 IU/mL) for 3 days. Replication-competent HIV NL4.3 virus pseudo-typed with VSV-G was used in the infection. The activated CD4^+^ T cells were infected with HIV-1 NL4.3 virus at MOI of 0.01 via spinoculation for 2 hours at 27°C as previously described. After infection, HIV-infected CD4^+^ T cells were washed and seeded in triplicate (1 × 10^5^ cells/well) in complete medium supplemented with 5% AB human serum and IL-2 (50 IU/mL) in 96-well plates. Primary CD8^+^ TCR transductants were added at E/T ratios of 1:1 and 5:1. An irrelevant TCR transductant (ECOV2) was included as control condition. After 5 days of coculture, cells were collected and stained with Live/Dead Fixable Aqua dye and flow antibodies targeting surface markers including anti-CD3-APC-Cy7 (300318, BioLegend), anti-CD8-BV421 (301036, BioLegend), and anti-CD4-PerCP-Cy5.5 (344607, BioLegend), followed by permeabilization using BD Biosciences fix/perm solution for intracellular staining of HIV Gag p24 antigen (KC57-FITC, 6604665, Beckman Coulter). Viral inhibition of HIV-infected cells was calculated as follows: percentage of inhibition on HIV infection = (percentage of HIV Gag-positive cells in CD4^+^ T cells cultured alone – percentage of Gag-positive cells in CD4^+^ T cells cultured with CD8^+^ TCR transductants)/percentage of Gag-positive cells in CD4^+^ T cells cultured alone) × 100. The viral suppression assay was performed with 3 replicates for each condition. Cells from each condition were pooled after culture to ensure more than 10,000 viable cells acquired for intracellular p24 staining and flow cytometry assessment.

### Statistics.

Data analysis was performed and graphs were generated using GraphPad Prism v10. A Mann-Whitney *U* test or unpaired 2-tailed *t* test was adopted to compare differences between 2 groups where applicable. A 1-way ANOVA followed by a multiple-comparison test was used to compare the differences among more than 2 groups. Statistical significance was defined as *P* < 0.05.

### Study approval.

Human peripheral blood mononuclear cells (PBMCs) were obtained from blood donated to NHS Blood and Transplant and from Cambridge Bioscience Ltd with ethical approval and written informed consent. They were processed, cultured, and cryopreserved in our laboratory under conditions licensed by the UK Human Tissue Authority.

### Data availability.

The raw data associated with the graphs in this paper are available in the Supporting Data Values file. Any further details can be obtained by direct contact with the corresponding authors.

## Author contributions

HS, HY, GMG, and AJM designed the study, performed experiments, analyzed data, and wrote the manuscript. PB and AEK were responsible for VSV pseudo-typed virus and infection protocols. SB, MNQ, and GMG contributed to HLA-E binding peptide prediction, SCT and DSF assays, and protein production. HS was responsible for the HLA-E peptide exchange assay. HS, HY, and WH conducted CL3 laboratory experiments. HS and MR conducted TCR cloning and transduction.

## Conflict of interest

The authors have declared that no conflict of interest exists.

## Funding support

This work is the result of NIH funding, in part, and is subject to the NIH Public Access Policy. Through acceptance of this federal funding, the NIH has been given a right to make the work publicly available in PubMed Central.

- Chinese Academy of Medical Sciences (CAMS) Innovation Fund for Medical Science (CIFMS), China (2024-I2M-2-001-1) (to AM).
- NIH National Institute of Allergy and Infectious Diseases UM1 AI 164567-02, the Collaboratory of AIDS Researchers for Eradication (CARE).
- China Scholarship Council-COI MD/PhD High-level Medical Innovative Talent Scholarship; Prevention and Control of Emerging and Major Infectious Diseases-National Science and Technology Major Project (2025ZD01904400); National Natural Science Foundation of China (82472269); and Non-profit Central Research Institute Fund of CAMS (2023-PT320-01) (to HS).
- National Natural Science Foundation of China (32500781) (to WH).

## Supplementary Material

Supplemental data

Supporting data values

## Figures and Tables

**Table 1:**
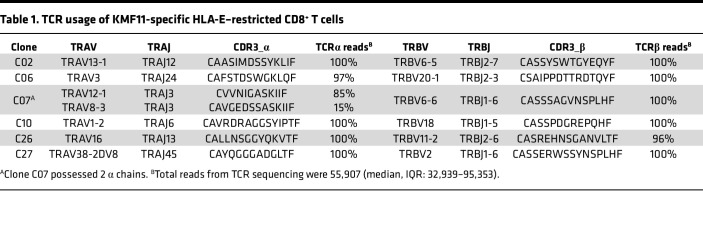
TCR usage of KMF11-specific HLA-E–restricted CD8^+^ T cells

## Version 1 05/19/2026

In-Press Preview

## Version 2 07/22/2026

Electronic publication

## References

[1] Braud VM (1998). HLA-E binds to natural killer cell receptors CD94/NKG2A, B and C. Nature.

[2] Heinzel AS (2002). HLA-E-dependent presentation of Mtb-derived antigen to human CD8+ T cells. J Exp Med.

[3] Joosten SA (2010). Mycobacterium tuberculosis peptides presented by HLA-E molecules are targets for human CD8 T-cells with cytotoxic as well as regulatory activity. PLoS Pathog.

[4] van Meijgaarden KE (2015). Human CD8+ T-cells recognizing peptides from Mycobacterium tuberculosis (Mtb) presented by HLA-E have an unorthodox Th2-like, multifunctional, Mtb inhibitory phenotype and represent a novel human T-cell subset. PLoS Pathog.

[5] Murugesan G (2024). Viral sequence determines HLA-E-restricted T cell recognition of hepatitis B surface antigen. Nat Commun.

[6] Burwitz BJ (2020). MHC-E-restricted CD8^+^ T cells target hepatitis B virus-infected human hepatocytes. J Immunol.

[7] Yang H (2023). HLA-E-restricted SARS-CoV-2-specific T cells from convalescent COVID-19 patients suppress virus replication despite HLA class Ia down-regulation. Sci Immunol.

[8] Wang EC (2002). UL40-mediated NK evasion during productive infection with human cytomegalovirus. Proc Natl Acad Sci U S A.

[9] Papadopoulos AO (2025). Spatial regulation of CD8^+^ T cells at the HLA-E-NKG2A axis drives HIV persistence in lymph node B cell follicles. Cell Rep.

[10] Früh K (2025). Targeting MHC-E as a new strategy for vaccines and immunotherapeutics. Nat Rev Immunol.

[11] https://www.unaids.org/en/resources/documents/2024/2024_unaids_data.

[12] Arunachalam PS (2020). T cell-inducing vaccine durably prevents mucosal SHIV infection even with lower neutralizing antibody titers. Nat Med.

[13] Borgo GM, Rutishauser RL (2023). Generating and measuring effective vaccine-elicited HIV-specific CD8 + T cell responses. Curr Opin HIV AIDS.

[14] (2024). Exploring synergies between B- and T-cell vaccine approaches to optimize immune responses against HIV-workshop report. NPJ Vaccines.

[15] Hansen SG (2013). Immune clearance of highly pathogenic SIV infection. Nature.

[16] Hansen SG (2016). Broadly targeted CD8^+^ T cell responses restricted by major histocompatibility complex E. Science.

[17] Malouli D (2022). Cytomegalovirus-vaccine-induced unconventional T cell priming and control of SIV replication is conserved between primate species. Cell Host Microbe.

[18] Yang H (2021). HLA-E-restricted, Gag-specific CD8^+^ T cells can suppress HIV-1 infection, offering vaccine opportunities. Sci Immunol.

[20] Bansal A (2021). HLA-E-restricted HIV-1-specific CD8+ T cell responses in natural infection. J Clin Invest.

[21] Stewart-Jones GB (2005). Structures of three HIV-1 HLA-B*5703-peptide complexes and identification of related HLAs potentially associated with long-term nonprogression. J Immunol.

[22] Gillespie GM (2002). Cross-reactive cytotoxic T lymphocytes against a HIV-1 p24 epitope in slow progressors with B*57. AIDS.

[23] O’Callaghan CA, Bell JI (1998). Structure and function of the human MHC class Ib molecules HLA-E, HLA-F and HLA-G. Immunol Rev.

[24] Walters LC (2018). Pathogen-derived HLA-E bound epitopes reveal broad primary anchor pocket tolerability and conformationally malleable peptide binding. Nat Commun.

[25] Walters LC (2020). Detailed and atypical HLA-E peptide binding motifs revealed by a novel peptide exchange binding assay. Eur J Immunol.

[26] Martinuzzi E (2011). acDCs enhance human antigen-specific T-cell responses. Blood.

[27] Stewart-Jones GB (2012). Structural features underlying T-cell receptor sensitivity to concealed MHC class I micropolymorphisms. Proc Natl Acad Sci U S A.

[28] Gillespie GM (2006). Strong TCR conservation and altered T cell cross-reactivity characterize a B*57-restricted immune response in HIV-1 infection. J Immunol.

[29] Sullivan LC (2017). A conserved energetic footprint underpins recognition of human leukocyte antigen-E by two distinct αβ T cell receptors. J Biol Chem.

[30] Walters LC (2022). Primary and secondary functions of HLA-E are determined by stability and conformation of the peptide-bound complexes. Cell Rep.

[31] Barber C (2022). Structure-guided stabilization of pathogen-derived peptide-HLA-E complexes using non-natural amino acids conserves native TCR recognition. Eur J Immunol.

[32] Kosmrlj A (2010). Effects of thymic selection of the T-cell repertoire on HLA class I-associated control of HIV infection. Nature.

[33] Chen H (2012). TCR clonotypes modulate the protective effect of HLA class I molecules in HIV-1 infection. Nat Immunol.

[34] Yu XG (2007). Mutually exclusive T-cell receptor induction and differential susceptibility to human immunodeficiency virus type 1 mutational escape associated with a two-amino-acid difference between HLA class I subtypes. J Virol.

[35] Turnbull EL (2006). HIV-1 epitope-specific CD8+ T cell responses strongly associated with delayed disease progression cross-recognize epitope variants efficiently. J Immunol.

[36] Mendoza JL (2020). Interrogating the recognition landscape of a conserved HIV-specific TCR reveals distinct bacterial peptide cross-reactivity. Elife.

[37] Aleksic M (2012). Different affinity windows for virus and cancer-specific T-cell receptors: implications for therapeutic strategies. Eur J Immunol.

[38] Bridgeman JS (2012). Structural and biophysical determinants of αβ T-cell antigen recognition. Immunology.

[39] Wooldridge L (2007). Enhanced immunogenicity of CTL antigens through mutation of the CD8 binding MHC class I invariant region. Eur J Immunol.

[40] Wooldridge L (2005). Interaction between the CD8 coreceptor and major histocompatibility complex class I stabilizes T cell receptor-antigen complexes at the cell surface. J Biol Chem.

[41] Rius C (2018). Peptide-MHC class I tetramers can fail to detect relevant functional T cell clonotypes and underestimate antigen-reactive T cell populations. J Immunol.

[42] Dolton G (2018). Optimized Peptide-MHC multimer protocols for detection and isolation of autoimmune T-cells. Front Immunol.

[43] Cole DK (2007). Human TCR-binding affinity is governed by MHC class restriction. J Immunol.

[44] (2011). High prevalence of low affinity peptide-MHC II tetramer-negative effectors during polyclonal CD4+ T cell responses. J Exp Med.

[45] Wooldridge L (2009). Tricks with tetramers: how to get the most from multimeric peptide-MHC. Immunology.

[46] Massilamany C (2011). Detection of autoreactive CD4 T cells using major histocompatibility complex class II dextramers. BMC Immunol.

[47] Singhaviranon S (2025). Low-avidity T cells drive endogenous tumor immunity in mice and humans. Nat Immunol.

[48] Scriba TJ (2005). Ultrasensitive detection and phenotyping of CD4+ T cells with optimized HLA class II tetramer staining. J Immunol.

[49] Schrum AG (2003). Surface T-cell antigen receptor expression and availability for long-term antigenic signaling. Immunol Rev.

[50] Gao F (2023). Spheromers reveal robust T cell responses to the Pfizer/BioNTech vaccine and attenuated peripheral CD8^+^ T cell responses post SARS-CoV-2 infection. Immunity.

[51] Zehn D (2009). Complete but curtailed T-cell response to very low-affinity antigen. Nature.

[52] Hay ZLZ (2023). Low TCR binding strength results in increased progenitor-like CD8+ tumor-infiltrating lymphocytes. Cancer Immunol Res.

[53] Denkberg G (2001). Critical role for CD8 in binding of MHC tetramers to TCR: CD8 antibodies block specific binding of human tumor-specific MHC-peptide tetramers to TCR. J Immunol.

[54] Janicki CN (2008). Loss of CTL function among high-avidity tumor-specific CD8+ T cells following tumor infiltration. Cancer Res.

[55] Wahl A, Garcia JV (2025). Humanized mouse systems to study viral infection: a new era in immunology research. Annu Rev Immunol.

[56] Lampen MH (2013). Alternative peptide repertoire of HLA-E reveals a binding motif that is strikingly similar to HLA-A2. Mol Immunol.

