# Supplementary material for: HLA-E–restricted T cells primed by a modified HLA-B*57:01–restricted HIV-1 peptide suppress HIV-1 replication
Source: JCI Insight. 2026 May 19;11(14):e203593. doi: 10.1172/jci.insight.203593 (PMC13461157; doi:10.1172/jci.insight.203593)
Supplement: Supplementary file 1 [file jciinsight-11-203593-s052.pdf]

Supplementary Figures and Tables:

Table S1. HLA typing of two healthy donors.

HLA type	Donor 1	Donor 2
HLA-A	A01:01/04N; A24:02	A25:01; A68:01
HLA-B	B44:05; B52:01	B44:02
HLA-C	Cw02:02; Cw12:02	Cw05:01/03; Cw07:04/11
HLA-E	01:01; 01:03	01:01; 01:03

Figure S1

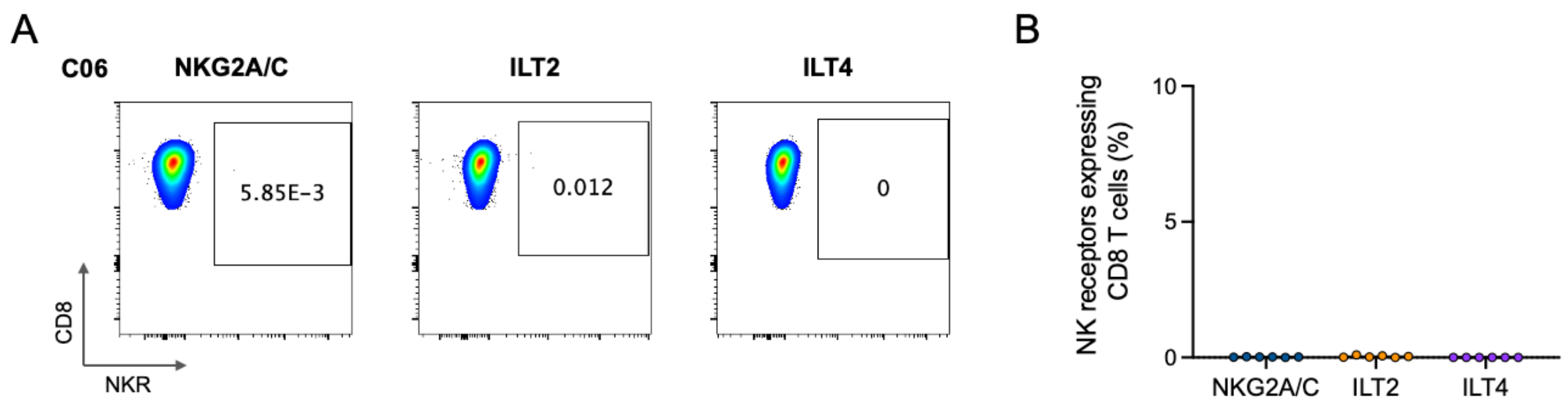

Fig. S1. Identification of VL9 specificity and expression of NKG2A/C, ILT2 and ILT4 of all the T cell clones generated.

A, *Representative flow cytometry plots demonstrating the low level or lack of NK receptors (NKG2A/C, ILT2 and ILT4) staining on a T cell clone C06.*

B, *NKG2A/C, ILT2, and ILT4 expression demonstrated minimal expression of these receptors on all 6 clones, including clones C02, C06, C07, C10, C26, C27.*

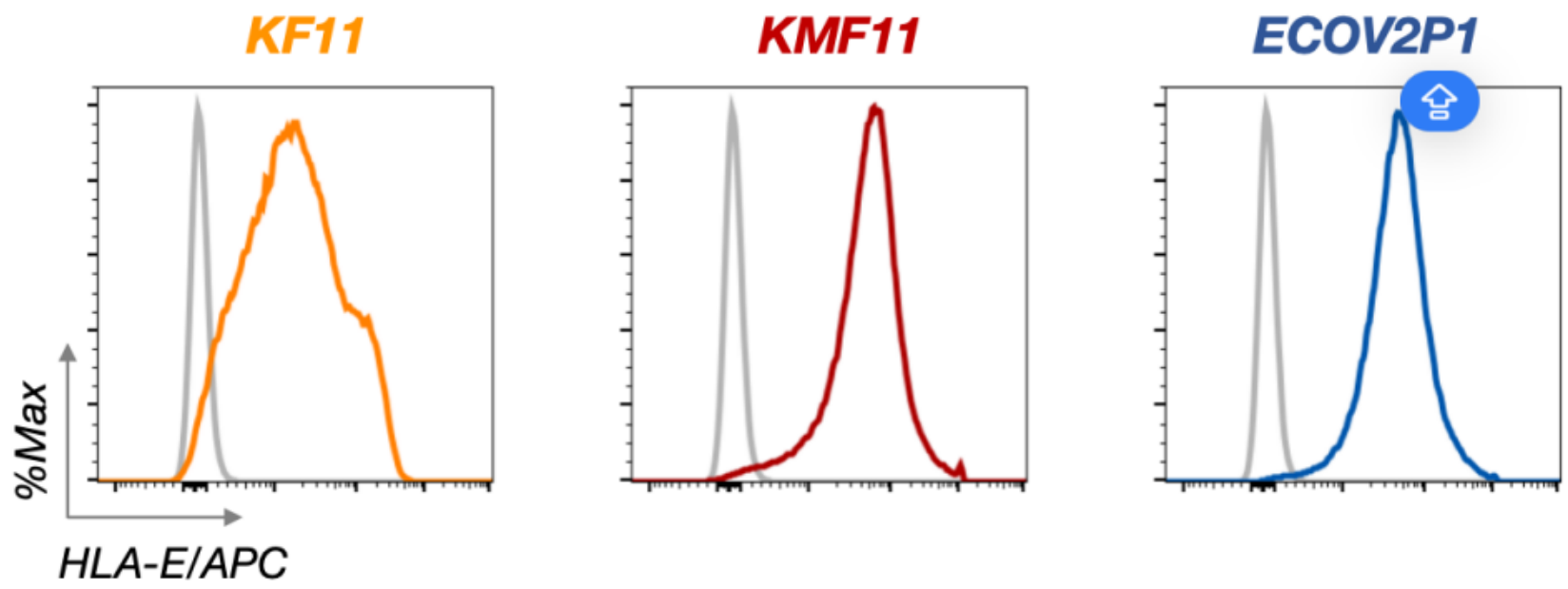

Fig S2. Generation of HLA-E SCT transduced K562 cells as antigen presenting cells
The flow charts represent the stable expression HLA-E in K562 cells transduced with SCTs containing the peptides KF11, KMF11 and ECOV2P1.

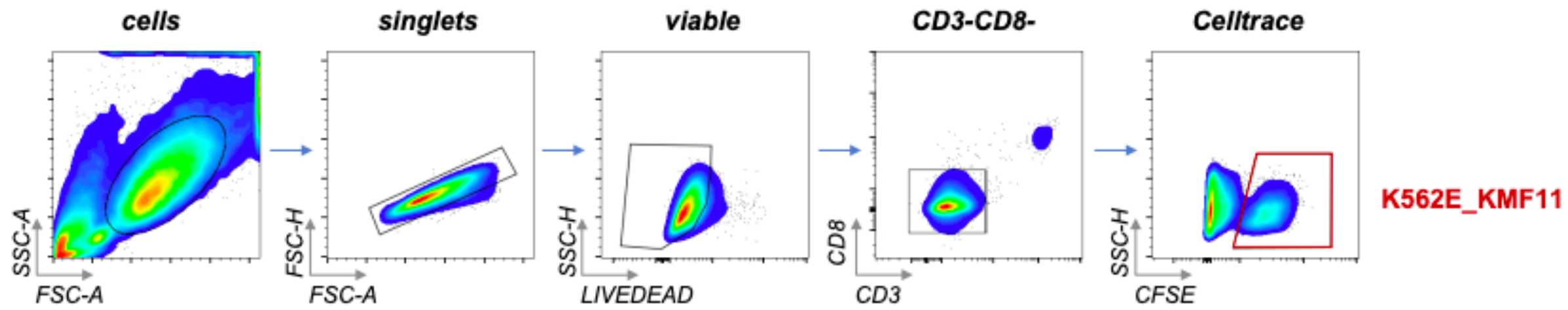

Fig. S3. Gating strategy of coculture assays with K562E target cells

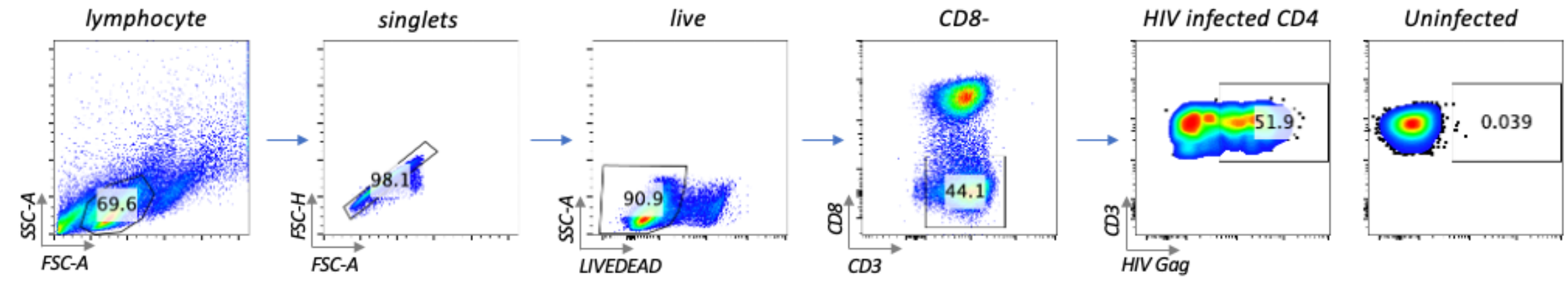

Fig. S4. Gating strategy of HIV-infected CD4 T cells in a viral suppression assay

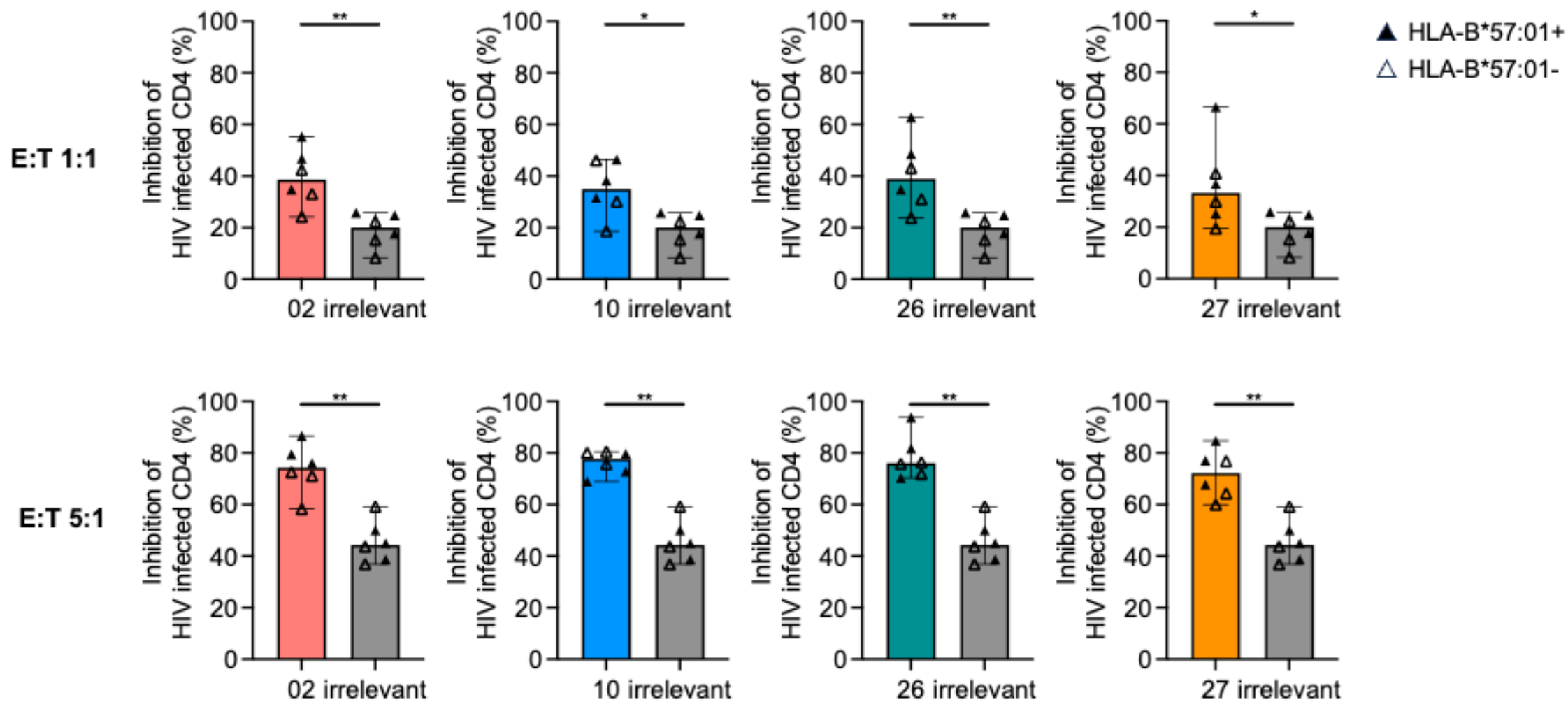

Fig. S5. Antiviral effect of KMF11-HLA-E-specific TCR CD8 T cell transductants against HIV-infected primary CD4 T cells in a viral suppression assay.

*The inhibitory effect of KMF11-specific TCR transductants on HIV-infected CD4 T cells was assessed at E:T ratios of 1:1 and 5:1, shown in another two independent experiments. Data are represented as the median with a 95% Confidence Interval (CI) (n=6 donors). Statistical significance between KMF11 TCR transductants and irrelevant TCR controls was determined using the Mann Whitney test (*p < 0.05, **p < 0.01). Solid triangle indicates HLA-B*57:01+ CD4 T cell donors, open triangle indicates HLA-B*57:01- CD4 T cell donors.*
